# Application of a bioinformatic pipeline to RNA-seq data identifies novel virus-like sequence in human blood

**DOI:** 10.1093/g3journal/jkab141

**Published:** 2021-04-29

**Authors:** Marko Melnick, Patrick Gonzales, Thomas J LaRocca, Yuping Song, Joanne Wuu, Michael Benatar, Björn Oskarsson, Leonard Petrucelli, Robin D Dowell, Christopher D Link, Mercedes Prudencio

**Affiliations:** 1 Department of Integrative Physiology, University of Colorado, Boulder, CO 80303, USA; 2 Department of Health and Exercise Science, Center for Healthy Aging, Colorado State University, Fort Collins, CO 80523, USA; 3 Department of Neuroscience, Mayo Clinic, Jacksonville, FL 32224, USA; 4 Department of Neurology, University of Miami, Miami, FL 33136, USA; 5 Department of Neurology, Mayo Clinic, Jacksonville, FL 32224, USA; 6 Neuroscience Graduate Program, Mayo Clinic Graduate School of Biomedical Sciences, Jacksonville, FL 32224, USA; 7 BioFrontiers Institute and Department of Molecular, Cellular and Developmental Biology, University of Colorado, Boulder, CO 80303, USA; 8 Institute for Behavioral Genetics, University of Colorado, Boulder, CO 80303, USA

**Keywords:** ALS, transcriptomics, RNA-seq, microbiome, virome

## Abstract

Numerous reports have suggested that infectious agents could play a role in neurodegenerative diseases, but specific etiological agents have not been convincingly demonstrated. To search for candidate agents in an unbiased fashion, we have developed a bioinformatic pipeline that identifies microbial sequences in mammalian RNA-seq data, including sequences with no significant nucleotide similarity hits in GenBank. Effectiveness of the pipeline was tested using publicly available RNA-seq data and in a reconstruction experiment using synthetic data. We then applied this pipeline to a novel RNA-seq dataset generated from a cohort of 120 samples from amyotrophic lateral sclerosis patients and controls, and identified sequences corresponding to known bacteria and viruses, as well as novel virus-like sequences. The presence of these novel virus-like sequences, which were identified in subsets of both patients and controls, were confirmed by quantitative RT-PCR. We believe this pipeline will be a useful tool for the identification of potential etiological agents in the many RNA-seq datasets currently being generated.

## Introduction

As detailed below, there are numerous reports suggesting that microbes could play a role in neurodegenerative diseases. Microbial sequences are routinely identified in human RNA-sequencing (RNA-seq) data (Mangul *et al.* 2018), which is typically acquired to assay gene expression. The origins of these microbial sequences are generally unknown, although in theory disease-relevant microbes could be identified if their sequences are significantly enriched in patients compared with controls. We therefore sought to develop a bioinformatic pipeline that could identify microbial sequences over-represented in RNA-seq data from patients compared with controls. Importantly, our pipeline can recover both known and novel microbial sequences.

### Background of organisms in neurodegeneration

Infection has been proposed to play a role in multiple neurodegenerative diseases (Patrick *et al.* 2019), including amyotrophic lateral sclerosis (ALS) (Castanedo-Vazquez *et al.* 2019). ALS is the most common motor neuron disease in adults, with the majority of individuals dying within 3–5 years of symptom onset. The disease is defined by the degeneration and death of motor neurons in the brain and spinal cord, resulting in progressive weakness and eventually death, typically from respiratory muscle weakness (Mehta *et al.* 2018). Around 10% of ALS patients have a family history that suggests an autosomal dominant inheritance which is classified as familial ALS (fALS), with the remaining 90% of patients classified as having sporadic ALS (sALS; Masrori and Van Damme 2020). After decades of study, the etiology of sALS remains a mystery, although suspected risk factors for ALS include exposure to heavy metals, pesticides, chemical solvents, cigarette smoke, and unidentified factors related to US military service (Ingre *et al.* 2015; Talbott *et al.* 2016; Zhan and Fang 2019; Opie-Martin *et al.* 2020). Along with these environmental risk factors, there has been a long history, with variable success, in the search for pathogens that might contribute to ALS (Pertschuk *et al.* 1977; Kohne *et al.* 1981; Alonso *et al.* 2017; Xue *et al.* 2018; Andrade *et al.* 2019) and other neurodegenerative diseases such as Alzheimer’s disease (AD; Deutsch *et al.* 1982; Taylor *et al.* 1984; Sochocka *et al.* 2017), Parkinson’s disease (PD; Irkeç 1982; Abushouk *et al.* 2017; Parashar and Udayabanu 2017), and multiple sclerosis (MS; Libbey *et al.* 2014).

Studies on ALS primarily come from European populations and within these populations four genes [TAR DNA-binding protein 43 (TDP-43), fused in sarcoma/translocated liposarcoma (FUS), superoxide dismutase 1 (SOD1), *chromosome 9 open reading frame 72* (*C9ORF72*)] account for 70% of fALS (Kiernan *et al.* 2011). Of these four genes, *C9ORF72* accounts for up to 30–50% of cases in fALS and 7% of sALS (in all populations; Masrori and Van Damme 2020). In *C9ORF72*-associated ALS (c9ALS), a hexanucleotide repeat expansion (HRE) occurs that can form RNA with highly stable parallel G-quadruplex structures (G4 RNA). How neurodegeneration occurs from HRE in c9ALS is not well understood, but putative mechanisms include reduction of *C9ORF72* expression, production of poly-dipeptides as a result of repeat-associated non-AUG translation of repeat sequences, and the formation of RNA foci that may sequester RNA binding proteins (Reddy *et al.* 2013; Tang *et al.* 2020). Identifying disease modifiers is of significant translational interest, as it is currently unknown how patients with c9ALS (sporadic or familial) progress from asymptomatic to symptomatic states. Evidence is mounting that persistent immune activation can play a causative roll in disease progression, and some recent treatments focus on reducing the elevated neuroinflammation that occurs in patients with the HRE (Trageser *et al.* 2019). Indeed, one study showed that a lower abundance of immune-stimulating bacteria contributes to reduced inflammation and protection from premature mortality in a *C9orf72* loss-of-function mouse model (Burberry *et al.* 2020).

Diverse pathogens have been reported in the blood, cerebrospinal fluid (CSF), and central nervous system (CNS) from ALS patients. For example, bacteria that have been detected include *Cutibacterium acnes, Corynebacterium* sp*, Fusobacterium nucleatum, Lawsonella clevelandesis*, and *Streptococcus thermophilus* in CSF (Alonso *et al.* 2019), and mycoplasma in blood (Gil *et al.* 2014). Fungi, including *Candida famata*, *Candida albicans*, *Candida parapsilosis*, *Candida glabrata*, and *Penicillium notatum*, have been detected in CSF, whereas *Malassezia globosa*, *Cryptococcus neoformans* (Alonso *et al.* 2017), and *C. albicans* have been found in various regions of the CNS (Alonso *et al.* 2015, 2017; Pisa *et al.* 2016). The search for viruses that contribute to ALS pathology is much more extensive and includes studies on herpes virus (Pertschuk *et al.* 1977; Cermelli *et al.* 2003), enterovirus (Pertschuk *et al.* 1977; Berger *et al.* 2000; Giraud *et al.* 2001; Vandenberghe *et al.* 2010; Xue *et al.* 2018), human immunodeficiency virus (HIV; Verma and Berger 2006; Moodley *et al.* 2019), and human endogenous retrovirus (Douville *et al.* 2011; Li *et al.* 2015; Arru *et al.* 2018). Importantly, multiple studies using immunohistochemistry have shown an increased load of various pathogens in ALS samples compared with controls in multiple tissues suggesting these pathogens are present and cannot be simply attributed to contamination (Pertschuk *et al.* 1977; Alonso *et al.* 2015, 2017, 2019; Pisa *et al.* 2016). Ultimately, the presence of ALS dysbiosis is unresolved and remains an active area of investigation with evidence for (Fang *et al.* 2016; Zhang *et al.* 2017; Blacher *et al.* 2019; Sun *et al.* 2019; Obrenovich *et al.* 2020) and against it (Brenner *et al.* 2018).

The biological role that these alternative microbiotas play in ALS is also unclear. ALS patients may have a compromised blood brain barrier or blood spinal cord barrier function (Henkel *et al.* 2009; Garbuzova-Davis and Sanberg 2014). It has been reported that ALS patients also have elevated Gram negative endotoxin/lipopolysaccharide (LPS) in the blood (Zhang *et al.* 2009). Patients with ALS also display activation of the innate immune system along with changes in blood (Mantovani *et al.* 2009; Murdock *et al.* 2017), spinal cord and motor neurons (Sta *et al.* 2011), but if and how bacteria might influence activation is an active area of research. A “dual hit” hypothesis by Correia *et al.* (2015) suggests inflammation via LPS may contribute to mislocalization and aggregation of ALS-implicated protein TDP-43.

Numerous studies have looked for biomarkers of ALS (Verber *et al.* 2019) using metabolomics (Blasco *et al.* 2010, 2017), neuroinflammation (Mitchell *et al.* 2009; Guo *et al.* 2017), DNA methylation (Young *et al.* 2017; Coppedè *et al.* 2018), gene expression (Swindell *et al.* 2019), microRNA expression (Waller *et al.* 2017, 2018) and our previous study which analyzed protein levels of poly(GP) in c9ALS (Gendron *et al.* 2017). The search for pathogens using sequencing data from blood samples in ALS patients has been conducted before (Gagliardi *et al.* 2018; van Rheenen *et al.* 2018; Rahman *et al.* 2019; Zucca *et al.* 2019), but previous efforts have not looked for novel pathogens. Next-generation sequencing (NGS) technologies have shown broad detection of pathogens in a target-independent unbiased fashion (Moore *et al.* 2011; Bouquet *et al.* 2017; Parker and Chen 2017; Westermann *et al.* 2017), however, designing a microbial detection experiment is nontrivial considering the variety of methods (Poussin *et al.* 2018) and algorithms (Roumpeka *et al.* 2017) that can be applied. Our primary goal when designing a new pipeline was to be conservative and unbiased with regards to discovery and quantification of novel pathogens. Furthermore, our intention was not to “reinvent the wheel” for microbiota classification, and instead opt to provide an end-to-end pipeline that leverages data across samples to obtain biologically significant fold changes of microbiota between diseased and healthy subjects.

Although other pipelines have used reads that do not map to the host genome (unmapped reads) for microbial identification and quantification, these pipelines cannot be used for discovery as they rely on existing databases of microbial genomes (Cavadas *et al.* 2017; Mangul *et al.* 2018; Simon *et al.* 2018; Gihawi *et al.* 2019). One popular pipeline for viral classification that uses nonhost reads includes ViromeScan (Rampelli *et al.* 2016), which utilizes a database of reference viral sequences to assign reads to taxonomic categories, but is “blind” to viral sequences not closely related to those in the database. Thus, we opted for de-novo assembly of unmapped reads into contigs, similar to the strategy employed by Kraken (Wood and Salzberg 2014) and MetaShot (Fosso *et al.* 2017). Additionally, we use a hierarchical method to assemble unmapped reads into contigs (single samples, group, and all) to increase the chance of assembling a correct contig from partial sequences that are present in multiple samples, and to remove outlier contigs present in single samples that are unlikely to contribute to the statistical analysis.

Where MetaShot stops at providing reads assigned to taxonomical categories, we map reads back to contigs and provide proper library normalization for statistical quantification. A similar pipeline known as IMSA (Cox *et al.* 2017) also maps reads back to contigs, but disregards contigs that might be identified by translated amino acid sequence similarity using BLASTX (a set we call the “dark biome”) as well as subsequent contigs with no BLASTN or BLASTX hit (a set we call the “double dark biome”).

We validated our pipeline by using datasets (synthetic and real) with known bacterial or viral infections. We also examined the differences in microbial identification between polyA and total RNA recovery in multiple tissues, and investigated the effects of globin depletion of blood samples. We then used our pipeline on a novel ALS blood dataset (termed “Our Study”) as well as on five other published ALS datasets from blood or spinal cord samples, analyzed each dataset individually, and analyzed across datasets for changes in microbiota. Although we did not identify any microbes enriched in the blood of ALS patients, we did identify and validate a novel virus-like sequence, demonstrating the potential of the bioinformatic pipeline we have established.

## Materials and methods

### Blood collection and RNA extraction

A total of 120 RNA whole blood samples constitute Our Study, which included 30 healthy controls (from general population that do not have blood relatives suffering from ALS, CTL), 30 presymptomatic *C9ORF72* mutant carriers (C9A), 30 symptomatic *C9ORF72* ALS cases (C9S), and 30 symptomatic *C9ORF72*-negative ALS cases (SYM). PAXgene blood RNA tubes were collected at Mayo Clinic Jacksonville and at University of Miami. All 120 RNA samples selected for RNA-seq were received and processed at Mayo Clinic Jacksonville using PAXgene blood RNA kit following manufacturer’s recommendations (Qiagen). Blood RNA was of high quality, assessed in an Agilent Bioanalyzer (Agilent), with RNA integrity values ranging from 7.4 to 9.8, with a median value of 8.7. RNA samples were then sent to The Jackson Laboratory for globin depletion, library preparation and sequencing of total blood RNA.

### Globin depletion

Due to the abundance of large hemoglobin RNA transcripts present in the blood, a globin depletion step, using the Ambion GLOBINclear kit (AM1980), was performed before sequencing of the blood RNA samples in order maximize coverage on nonglobin genes. In brief, one microgram of total RNA was used as starting material, and specific biotinylated oligos were used to capture globin mRNA transcripts. The capture oligos were hybridized with total RNA samples at 50°C for 30 min. Streptavidin magnetic beads were then used to bind to the biotinylated capture oligos hybridized to globin mRNA by incubating at 50°C for 30 min. The magnetic streptavidin beads-biotin complex were then captured to the side of the tubes by a magnet, and the resulting supernatant is free of globin mRNA. The globin depleted (GD) RNA was further purified by RNA binding beads and finally eluted in elution buffer. The resulting RNA free of >95% globin mRNA transcripts was then processed for NGS. Of note, to assess the efficiency of the globin RNA depletion, 10% of the samples processed were selected randomly and amplified using a Target-Amp Nano labeling kit (Epicentre). Samples were normalized to 100 ng input and reverse transcribed. First strand cDNA was generated by incubating at 50°C for 30 min with first strand premix and Superscript III. This was followed by second strand cDNA synthesis through DNA polymerase by incubating at 65°C for 10 min and at 80°C for 3 min. *In vitro* transcription was then performed at 42°C for 4 h followed by purification using RNeasy mini kit (Qiagen).

Due to the large number of samples, the globin depletion step was performed in two batches. We provided guidelines on how samples would be divided among the batches and also for how samples would be grouped in the sequencing runs in order to minimize technical variability. The Jackson Laboratory personnel were blinded to the identity of the samples.

RNA-seq of total blood RNA (globin and ribosomal RNA depleted) was performed in an Illumina HiSeq4000 with >70 million read pairs per sample (100 bp read lengths). Raw reads were then sent back to us for bioinformatics analyses.

### Quantitative RT-PCR for blood RNA samples

A total of 500 ng of total blood RNA was used for reverse transcription polymerase chain reaction (RT-PCR), using the high-capacity complementary DNA Transcription Kit with random primers (Applied Biosystems). Quantitative real-time PCR (qRT-PCR) was performed using SYBR GreenER qPCR SuperMix (Invitrogen). Samples were run in triplicate, and qRT-PCRs were run on a QuantStudio 7 Flex Real-Time system (Applied Biosystems).

List of primers and their sequences in this study:

Primers targeting the novel RNA-dependent RNA polymerase (RDRP) contig from our study

*RDRP* forward 5′-GCTGTCAAATCGGTTTCCAAC-3′;

*RDRP* reverse 5′-CTGCCTTCGTCATCTTGGAG-3′.

Primers targeting highly expressed control regions

*GAPDH* forward 5′-GTTCGACAGTCAGCCGCATC-3′;

*GAPDH* reverse 5′-GGAATTTGCCATGGGTGGA-3′.

### Transcriptomics

See pipeline description in results for an overview of the pipeline; see bioinformatics Supplementary File S1 for a more detailed description of the analysis pipeline, versions, and statistical quantification. For downloading the pipeline and detailed instruction for running the pipeline please read the README at https://github.com/Senorelegans/MysteryMiner. We have also deposited a frozen and cite-able version of the software with doi:10.5281/zenodo.4598807 and available at https://zenodo.org/record/4598807#.YEphh5NKjKp. All data in this study were processed identically using the pipeline.

### Statistical analysis

To assess statistical differences between conditions, a two tailed Student’s *t*-test was calculated using normalized coverage (NC) for contigs or binned normalized coverage (BNC) for species/genus, etc. The number of contigs or genus/species is used to obtain an False discovery rate (FDR) corrected (using the Benjamini/Hochberg method) adjusted *P*-value (*q*-value) via statsmodels in Python. Cutoff for statistical significance is less than an *q*-value of 0.05 unless otherwise stated.

## Results

### Pipeline description

**Figure 1:**
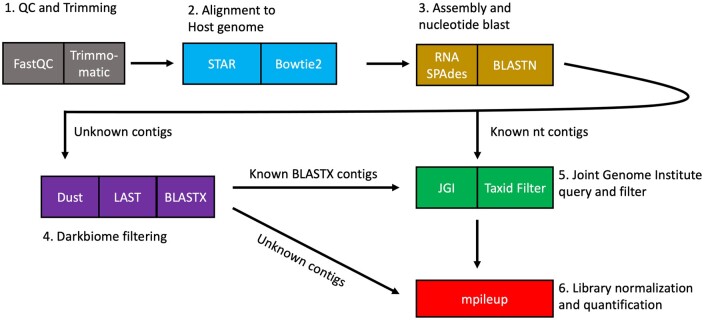
Diagram of Mystery Miner pipeline. Reads were first checked with FastQC and trimmed using Trimmomatic (1, gray). Reads were then aligned to the host genome using various aligners (2, blue). Nonhost (unmapped) reads were assembled into contigs with RNA SPAdes and regular biome contigs were identified with BLASTN (3, yellow). Unidentified contigs were filtered for repetitive sequences with Dust, filter by single, group or all with LAST, and dark biome contigs were identified with BLASTX. Double dark biome unidentified BLASTX contigs were sent directly to quantification (4, purple). Dark biome and regular biome contigs were assigned complete taxonomy using the JGI server and filtered one last time to remove mammalian/host genome contigs (5, Green). Nonhost reads were then mapped to all contigs and NC was calculated for subsequent statistical analysis.

Our novel pipeline, Mystery Miner, is written as a Nextflow pipeline. Below is a short overview of the Mystery Miner pipeline (Figure 1). A more in-depth explanation, list of software and versions used, and typical parameters of each step are described in the bioinformatics supplement, and all of the code used in this article can be found at https://github.com/Senorelegans/MysteryMiner.

Raw reads were first checked for quality using FastQC then trimmed to remove both adaptor contamination and low quality basecalls using Trimmomatic. Trimmed reads were then mapped to the host genome using STAR for a fast first-pass followed by a second pass with bowtie2 for sensitivity. Unmapped reads were retained for contig assembly. Filtering out host reads made downstream assembly faster and required less memory. We assembled contigs from unmapped reads with the SPAdes assembler (with “-rna” setting). This assembler was chosen for its recent use in studies of microbial diversity (Almeida *et al.* 2019) and proven robustness to biological and technical variation (Papudeshi *et al.* 2017). The species each contig belongs to was identified with BLASTN using default settings, and the top hit for each contig was retained (a set we call “regular biome”). Contigs with no BLASTN hits were then filtered to remove highly repetitive regions (DUST). Next, contigs were retained if they had a >60% pairwise alignment (LAST) between contigs assembled from a single sample, group/condition, or all samples (for example; contigs from groups that match singles are retained, we then use this new set to match with contigs from the all assembly).

We then identified contigs that lacked detectable nucleotide similarity to any GenBank entry but showed similarity at the amino acid level using BLASTX (“dark biome”). Furthermore, contigs with no BLASTN or BLASTX hits were labeled as “double dark biome” (also filtered by DUST and LAST). Every contig in the “regular biome” and “dark biome” were then queried against the Joint Genome Institute Server for additional taxonomic information. As Mystery Miner is an agnostic tool, it cannot distinguish between true tissue or cell-associated microbes and experimentally introduced contamination.

For quantification, we mapped the nonhost reads using Bowtie2 to the contigs obtained from SPAdes. Next, we mapped reads to contigs using samtools mpileup (default mapq score) to calculate the amount of reads over each base pair in a contig. We then calculated coverage on the contigs by summing all of the counts for each base pair in a contig and dividing by the length of the contig. We then calculated NC by library size using the number of mapped reads to the host genome. This gave us NC for a contig or BNC for multiple contigs within a species/genus, etc. To assess statistical differences between conditions, a Student’s *t*-test was calculated through NC or BNC, using the number of contigs or genus/species to obtain an FDR corrected adjusted *P*-value (*q*-value) using statsmodels in Python.

### Validating Mystery Miner on datasets with known bacterial or viral infection

To confirm that Mystery Miner is able to recover and quantify known bacterial infections from sequencing data, we utilized an *in vitro* model of *Chlamydia trachomatis* infection from (Humphrys *et al.* 2013). In this study, epithelial cell monolayers were infected with *C. trachomatis*; and polyA RNA (six samples) and total RNA (six samples) were sequenced 1 and 24 h postinfection (hpi). Using the Mystery Miner pipeline, out of 5.32 × 10^6^ reads from all of the samples, 6.04 × 10^5^ reads remained unmapped (∼11.34%) after trimming and mapping to the host genome (Supplementary File S2). From these nonhost reads, 3257 contigs were assembled and 1199 of these contigs were identified as regular biome (Supplementary File S3). An additional 27 contigs had no BLASTN hit. Of these, we identified two dark biome (BLASTX identified) and no double dark biome (no BLASTX or BLASTN hit) contigs (Supplementary Files S4 and S5).

**Figure 2:**
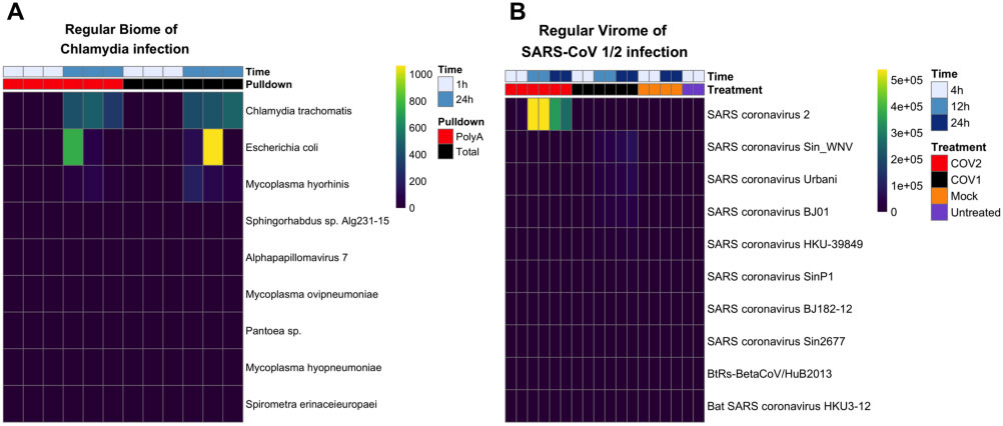
Heatmap of BNC for bacterial or viral infected datasets. (A) Regular biome contigs binned by species from Humphrys *et al.* (2016). Time refers to 1 or 24 hpi of epithelial cell monolayers with *C. trachomatis* (blue). Pulldown refers to library enrichment for polyA RNA (red) or total RNA (black). (B) Regular virome of contigs binned by name from Emanuel *et al.* (2020) for SARS-CoV-2 infected cells (COV2) (red), or SARS-CoV-1-infected cells (COV1) (black), mock virus (orange), or untreated sample (purple). Time refers 4, 12, or 24 hpi of Calu3 cells with indicated virus (blue). Top 10 hits per experiment shown for brevity.

Using Mystery Miner we successfully identified, and found significantly elevated levels, of *C. trachomatis* (BNC by species) in 24 hpi samples compared with 1 hpi samples in both polyA (*q* = 0.004) and total RNA (*q* = 0.0005). In addition to *C. trachomatis*, we identified six additional bacterial species and one viral species (Alphapapillomavirus 7) in the samples (Figure 2A), including significantly elevated levels of *Mycoplasma hyorhinis* contigs in total RNA samples. No significant differences were observed in the dark or double dark contigs (Supplementary File S6).

To confirm that the pipeline can detect known viral infections, we ran Mystery Miner on a total RNA dataset from an *in vitro* model of severe acute respiratory syndrome coronavirus (SARS-CoV)-1 or -2 infection (Emanuel *et al.* 2020). In this study, human epithelial Calu3 cells were infected with SARS-CoV-1 or SARS-CoV-2 (4, 12, or 24 h), mock (4 or 24 h), or untreated (4 h).

Out of the 2.81 × 10^8^ reads obtained from all of the samples, 8.23 × 10^7^ reads remained unmapped (∼29%) after trimming and mapping to the host genome (Supplementary File S2). From these nonhost reads, 42,816 contigs were assembled, of which 1346 regular biome, 27 dark biome, and 7 double dark biome contigs passed the filtering steps (Supplementary Files S2–S5).

Mystery Miner successfully identified both SARS-CoV-2 and SARS-CoV-1 isolates and found significantly elevated levels of each virus compared with controls (Figure 2B). Hereafter, we refer to SARS-CoV-1 or SARS-CoV-2-infected cells as COV1 or COV2 to avoid confusion with recovered names of contigs. Consistent with the viruses being similar, we identified both SARS-CoV-2 and SARS-CoV-1 in both the COV1-24hr and COV2-24hr samples when compared with mock-24hr. However, when we compared COV2-24hr to COV1-24hr, we were able to distinguish SARS-CoV-1 isolates from SARS-CoV-2 in the appropriate samples (*i.e.*, SARS-CoV-2 was significantly elevated in COV2). Similar results were seen in the 12 h samples but the 4 h samples did not have sufficient viral reads to detect either SARS-CoV virus (Supplementary File S7). To simulate a novel virus, we ran Mystery Miner on versions of the BLASTN and BLASTX databases obtained before SARS-CoV-2 was discovered and were able to properly identify SARS-CoV-2 as a bat-related CoV (Boni *et al.* 2020; Supplementary Figure S1 and File S7).

Collectively, these data show that Mystery Miner is able to identify potential bacterial and viral infections, properly identify infected samples using quantification, and detect significant differences between infected samples and controls for bacteria, viruses, and isolates of a virus.

### Validating Mystery Miner on a synthetic minibiome

We next looked at the detection and quantification limits of Mystery Miner using generated read data to create a synthetic minibiome. We used Polyester (Frazee *et al.* 2015) to generate paired end read data (100 bp read size) at various coverage levels and various fold change differences between two groups (groups A and B) with 10 samples each (20 samples total, read S1 for methods). Our synthetic minibiome consists of 10 human sequences and 10 sequences from nonhuman organisms (4 pathogenic and 6 commensal). The first four organisms in the synthetic minibiome are SARS-CoV-1, SARS-CoV-2, *C. trachomatis*, and *Chlamydia pneumoniae*. The next six (*Mageeibacillus indolicus*, *Prevotella melaninogenica*, *Filifactor alocis, Mobiluncus curtisii, Rothia dentocariosa*, and *Aeromicrobium marinum*) are commensals that are part of the representative bacteria list from the Human microbiome project (Ribeiro *et al.* 2012).

For the human sequences, we first generated a pool of human reads using the first 10 kb of 10 scaffolds from chromosome 22 (default value for human read generation in Polyester) at 1000× coverage with no fold change differences between groups. For nonhuman organisms, we took the first 10 kb of the nucleotide sequence for the organism and generated reads at coverage levels of 1000×, 100×, 10×, 1×, 0.1×, and 0.01×. Last, we combined the 1000× coverage human reads separately with each level of coverage for nonhuman organisms and ran Mystery Miner (six pipeline runs in total).

We found sequences below 1× coverage did not assemble, suggesting that this is our limit of detection (all further data omits 0.1× and 0.01× coverage). For the SARS strains, we successfully identified both strains at 1000× coverage but found that with lower coverage levels, SARS-CoV-1 was identified as a SARS-related CoV. This ambiguity is likely due to the 73% nucleotide sequence identity (aligned with CLUSTAL OMEGA; Ninfali 2003) between the first 10 kb of SARS-CoV-1 and SARS-CoV-2. For the selected *Chlamydia* species (59% sequence identity of the first 10 kb) and the rest of the commensal bacteria, we were able to successfully assemble and correctly identify each species at every level of coverage.

**Figure 3:**
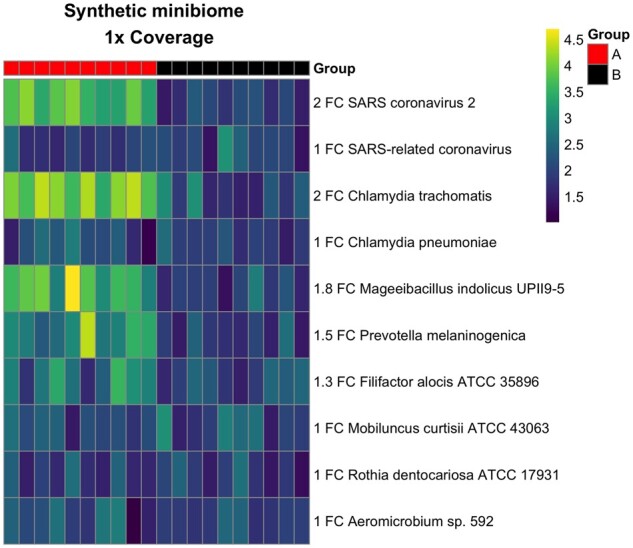
Heatmap of coverage of synthetic minibiome (1× coverage). Heatmap of coverage for synthetic minibiome at 1× coverage. Fold change (FC) in the row name refers to group A (red) over group B (black). The first four rows are pathogenic organisms, the next six rows are commensals identified from the human microbiome project.

Along with identification, we looked at Mystery Miners ability to quantify fold change differences between groups (A and B) using the synthetic minibiome. For the four pathogenic organisms, we selected one sequence from each kingdom to have a twofold difference (SARS-CoV-2, *C. trachomatis*). For the six commensals, we chose the first three species to have fold change differences of 1.8, 1.5, and 1.3 (*M. indolicus*, *P. melaninogenica*, and *F. alocis*). For SARS, we found that at 1× coverage, the twofold difference of SARS-CoV-2 was correctly called significant (*q* = 5.14 *e*^−10^), but the ambiguously identified SARS-related CoV contig was not called significant (*q* = 0.489). At 1000× coverage, we found that the correctly identified SARS-CoV-1 contig was falsely called significant (*q* = 0.0028), this is likely due to ambiguous read mapping from the closely related SARS-CoV-2 sequence, as mentioned above. We found similar results for each coverage level (from 1× to 1000×) for the rest of the organisms and will subsequently use values from 1× coverage as that is the lowest level of detection. For *Chlamydia*, we found Mystery Miner successfully called *C. trachomatis* significant (*q* = 3.57 *e*^−10^) and *C. pneumoniae* not significant (*q* = 0.709). For the commensals with FC differences, we successfully called each one significant [*M. indolicus* (*q* = 6.92 e^−7^), *P. melaninogenica (q = *4.91 e^−5^), *F. alocis* (*q = 0*.017); Figure 3 and Supplementary File S7]. Using synthetic data, we conclude that Mystery Miner is able to identify organisms down to the species level and correctly call significant fold changes at low levels of coverage but has difficulty from ambiguity when reads come from highly similar sequences (>72%).

### Effects of library pulldown or globin depletion in RNA-seq datasets

In order to compare effects of library enrichment or depletion, we compared recovered pathogens in a dataset that has polyA enrichment or rRNA depleted total RNA from blood or colonic tissue (Zhao *et al*. 2018). When we compared polyA RNA versus total RNA and looked at BNC by superkingdom of bacteria we found significantly more reads map to bacteria for total RNA than polyA RNA (*q* = 0.0349), in blood but not in colon (*q* = 0.11709; Supplementary Figure S2 and File S8). We found similar amounts of significant BNC by species for polyA RNA versus total RNA in blood (29) and in colon (26). We then looked at significant BNC by genus and found double the amount in blood (14) compared with colon (7), with only one significant genus (*Actinomyces*) found in both comparisons. We did not find any significant differences in coverage when we looked at the species, genus or superkingdom level for viruses (Supplementary File S8). We conclude that library enrichment with total RNA compared with polyA RNA increases bacterial recovery and diversity in blood.

We next looked at a RNA-seq dataset from whole blood with GD versus nonglobin depleted (NGD) total RNA (Shin *et al.* 2014). With BNC by superkingdom, we found significantly increased levels in GD versus not-depleted samples for both bacteria (*q* = 0.004; Supplementary Figure S3) and viruses (*q* = 0.030; Supplementary Figure S4). We also found significant differences in BNC by species (Supplementary Figure S5) or genus (Supplementary Figure S6) primarily from *Escherichia coli* with elevated levels in globin-depleted blood RNA. We did not find any significant differences when we looked for viruses at the species or genus level (Supplementary File S9).

### Analysis of our study

We used Mystery Miner on our novel RNA-seq dataset of GD and rRNA depleted total blood RNA from 120 individuals. These samples were from four subject groups including healthy control participants (CTL), ALS symptomatic *C9ORF72* negative patients (SYM), *C9ORF72* positive ALS symptomatic patients (C9S), and *C9ORF72* positive asymptomatic individuals (C9A).

The entire dataset contains a combined 8.64 × 10^9^ reads. Approximately 2.7% (2.34 × 10^8^) of the reads did not map to the human genome. From these nonhost reads 2,976,988 contigs were assembled and 17,047 BLASTN contigs (regular biome) were identified. A total of 25,815 contigs had no BLASTN hit and after filtering we identified 2,980 dark biome (BLASTX identified) and 859 double dark biome (no BLASTX or BLASTN hit) contigs (Supplementary Files S2–5).

**Figure 4:**
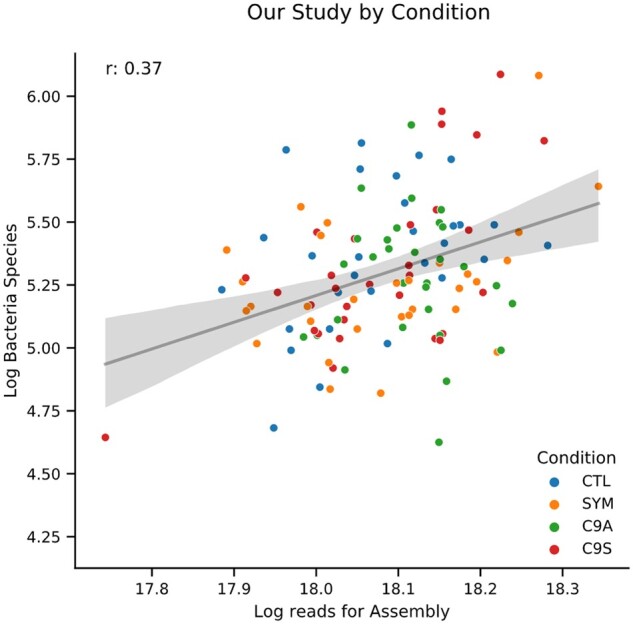
Log number of bacterial species versus Log reads for Assembly in Our Study. Scatterplot where each dot is a sample from a dataset with log number of bacterial contigs assembled on the Y-axis and Log reads used in SPAdes on the X-axis. Samples show a modest correlation (Pearson’s *r* = 0.37) between library size and bacterial species recovered. Data fit with a regression (black line) and 95% CI (gray area).

In general, we found a modest positive correlation between library size and number of bacterial contigs assembled, species detected (Figure 4), and genera detected for each sample as well as a homogenous mixture of samples with respect to disease status. No specific taxonomy or contig sequence correlated with participant class within the dataset. By pooling bacterial read counts across all of the samples, we found *alpha proteo-bacteria*, *Actinobacteria, Firmicutes*, and *Bacteroidetes* as the most highly represented taxonomies, consistent with other blood biome studies (Castillo *et al.* 2019; Supplementary Figure S7). Most of the bacterial genera (∼65%) assigned to the dark biome contigs were also found in the regular biome; however, this was not the case in the viral sets, as only 5% (4/78) of dark viral contigs were observed in the regular biome (Supplementary File S10). This observation suggested that our pipeline might be identifying novel viral sequences.

**Figure 5:**
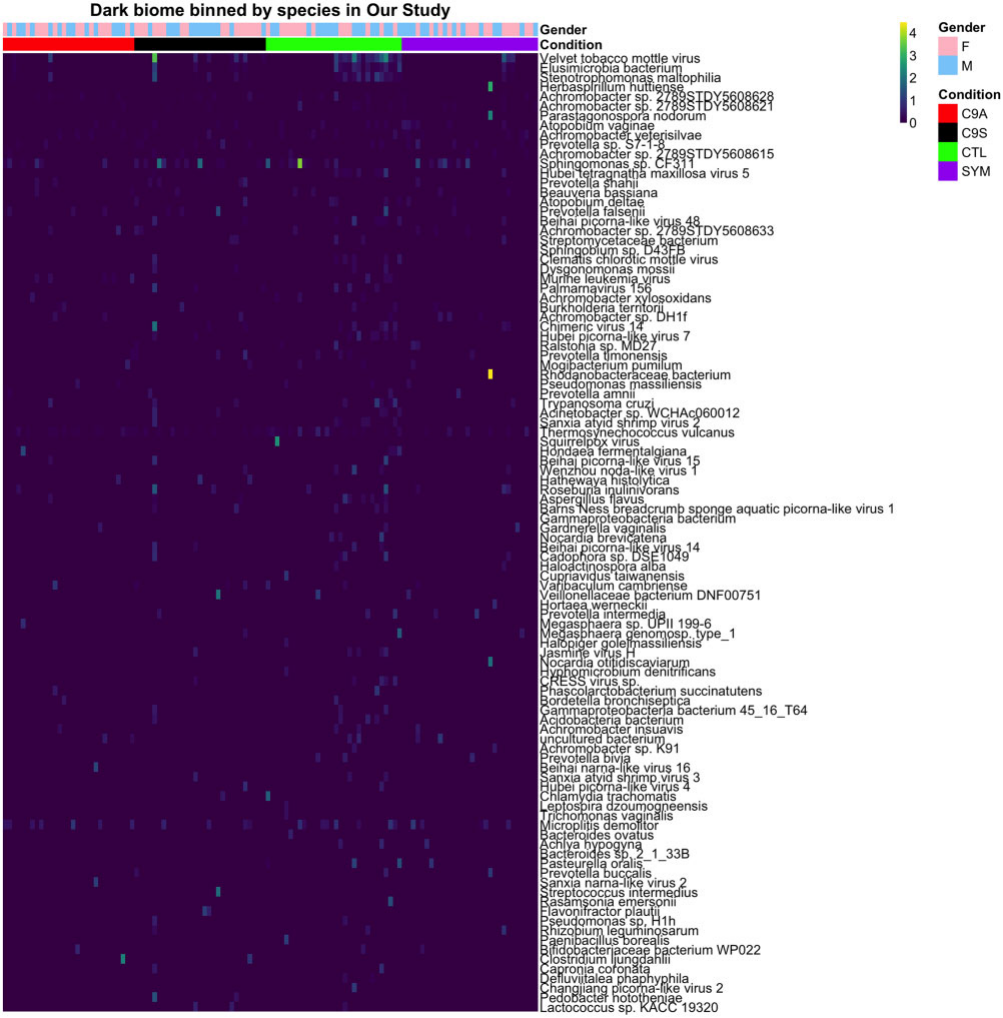
Heatmap of dark biome contigs binned by species in Our Study. Heatmap of NC of dark biome contigs binned by species. The highest coverage belongs to contigs that show high similarity to velvet tobacco mottle virus. Zero coverage is dark blue and goes to yellow with increasing values. These samples were from four subject groups including healthy controls [(CTL) green], *C9ORF72* negative ALS symptomatic [(SYM) purple], *C9ORF72* positive ALS symptomatic [(C9S) blue], and *C9ORF72* positive asymptomatic [(C9A) red] patients. Sex indicated as light blue (male) and pink (female). Top 100 species sorted by BNC shown for brevity.

Within the dark biome contigs, we noted numerous contigs with a region of protein sequence similarity to RDRP from multiple RNA viruses, including the velvet tobacco mottle virus (first row in heatmap of Figure 5, complete metadata shown in Supplementary Figure S8). Our attention was drawn to the largest (∼5 kb) dark biome contig hereafter labeled as “RDRP contig.” This large contig showed no nucleotide sequence similarity to any sequence in GENBANK, and no protein sequence similarity except for a long open reading frame with significant similarity to viral RDRPs (BLASTX *P* = 1*e*^−26^). A phylogeny based solely on viral RDRP protein sequences places the RDRP contig closest to single-stranded (+) viruses of the *Barnavirus*, *Sobemovirus*, and *Polerovirus* genera (Supplementary Figure S9 and File S1 for methods). However, given the absence of detectable similarity in this contig to other (non-RDRP) viral proteins of these genera, the relationship of the contig sequence to other virus groups is unclear, which supports the view that this contig represents a novel viral sequence.

To confirm the presence of the RDRP contig in the original samples, we designed primers to the RDRP contig and performed RT-PCR on seven samples, four of which had high coverage (predicted present) and three with zero coverage (predicted absent). We found typical levels for detection of a virusin the samples with high coverage and detected no signal above background in samples with zero coverage (Table 1). We conclude that Mystery Miner can recover true novel sequences that could represent previously unknown pathogens.

**Table 1:** RT-PCR and NC results for RDRP contig

Condition	Sample	GAPDH	RDRP	RDRP
		RT-PCR Ct value	RT-PCR Ct value	RNA-seq NC
SYM	LP00274	20.562019	36.401	1.56
C9S	LP00041	20.783213	36.346	3.39
C9S	LP00192	20.899612	35.636	0.67
C9A	LP000180	19.982108	34.832	1.11
C9S	LP000183	20.176418	Undetermined	0
C9S	LP000197	20.125161	Undetermined	0
C9A	LP000157	20.062433	Undetermined	0

qRT-PCR and NC results from the 5180 bp RDRP contig. For the RDRP contig positive samples (top 4) with high NC and detectable Ct values and negative samples (bottom 3) with no NC and undetectable Ct values. GAPDH was used as a positive control for qRT-PCR and shows comparable levels for all samples. These samples were from three conditions *C9ORF72* negative ALS symptomatic patients (SYM), *C9ORF72* positive ALS symptomatic patients (C9S), and *C9ORF72* positive asymptomatic individuals (C9A).

### Analysis of published ALS datasets

**Figure 6:**
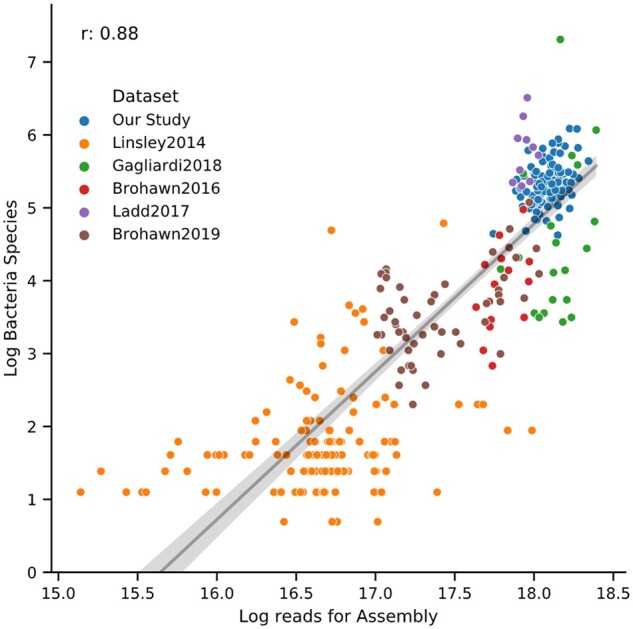
Log number of bacterial species versus Log reads for Assembly for ALS Datasets. Scatterplot where each dot is a sample from a dataset with log number of bacterial contigs assembled on the Y-axis and Log reads used in SPAdes on the X-axis. ALS datasets show a high correlation (Pearson’s *r* = 0.88) between library size and bacterial species recovered. Data fit with a regression (black line) and 95% CI (gray area).

We next sought to explore whether similar results would be obtained from other ALS datasets. To this end, we examined five other publicly available ALS datasets, consisting of two that used total RNA from blood (Linsley *et al.* 2014; Gagliardi *et al.* 2018) and three datasets from spinal cord (Brohawn *et al.* 2016, 2019; Bennett *et al.* 2019). We have provided a summary table of datasets for all studies used in this article (Table 2). As we observed in Our Study, we first noted that increased library size correlated with an increased number of bacterial contigs assembled, species detected, and genera detected (Figure 6 and Supplementary Figure S10–12 show all datasets used in this study).

**Table 2:** Study design for datasets used in this article

Name	Groups	No. of Samples	Tissue	Pulldown
Humphrys *et al.* (2013)	1 or 24 hpi with *C. trachomatis*	12	Cultured epithelial cell monolayers	PolyA Total RNA
Zhao *et al.* (2018)	PolyA or total RNA from blood or colon	16	Whole Blood	PolyA RNA
			Colon	Total RNA
Shin *et al.* (2014)	GD	24	Whole Blood	Total RNA
	NGD			
Emanuel *et al.* (2020)	SARS-CoV-1 or -2 infection	18	Calu3 cells	Total RNA
	Controls			
Our Study	*C9ORF72* negative ALS	120	Whole blood	Total RNA hemoglobin and rRNA depleted
	*C9ORF72* positive and symptomatic ALS			
	*C9ORF72* positive asymptomatic participants			
	Controls			
Linsley *et al.* (2014)	ALS	134	Whole blood	Total RNA
	Type 1 diabetes			
	Sepsis			
	MS patients before and 24 h after the first treatment with IFN-beta			
	Controls			
Gagliardi *et al.* (2018)	sALS	20	Peripheral blood mononuclear cells	Total RNA
	ALS with mutations in *FUS*, *SOD1*, and *TARDBP*			
	Controls			
Brohawn *et al.* (2016)	ALS	15	Cervical spinal cord	Total RNA
	Controls			rRNA depleted
Ladd *et al.* (2017)	ALS	10	LCM to isolate cervical spinal cord motor neurons	Total RNA
	Controls			
Brohawn *et al.* (2019)	ALS, AD, and PD	53	Cervical spinal cord	Total RNA
	Controls			

Overview of the datasets used in this article. The first three studies are only used to validate our pipeline. The six subsequent studies are ALS related from both blood and spinal cord.

**Figure 7:**
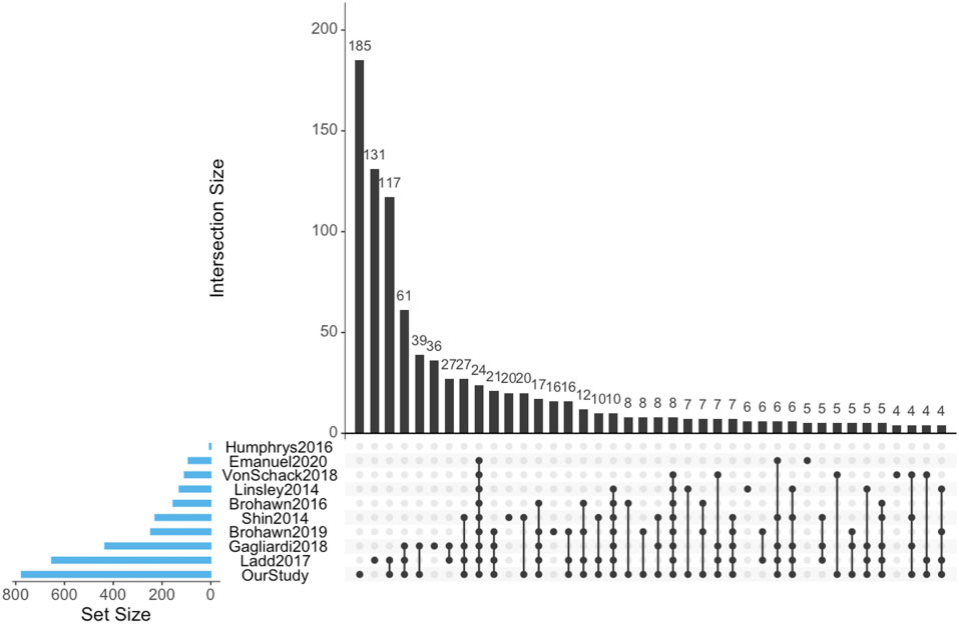
Upset plots of overlapping genus in the regular bacteriome between datasets. Upset plots are Venn diagram-like plots. A set refers to a dataset used in this study and each set is on a row with total amounts in a set as a blue bar plot on the left (ordered by set size). The black histogram on top shows the counts that are in the intersection of sets (a single dot for one dataset or connected dots for overlap of multiple datasets). Intersections less than four are removed for visualization purposes.

We then looked at the total overlap of genus or species to determine if there are similarities in recovered microbial or viral sequences between datasets. For genus in the regular bacteriome, our dataset had the highest number of unique genus (185), followed by Ladd *et al.* (2017; 117), and Gagliardi *et al.* (2018; 38). The highest number of overlapping bacterial genus was between our dataset and Ladd *et al.* (2017; 133) followed by the intersection between our dataset (Ladd *et al.* 2017; Gagliardi *et al.* 2018; 61) and there was a modest overlap (24) for 9/10 datasets (Figure 7). We observed roughly the same trend in the regular bacterial biome at the species level and in the dark bacterial biome (Supplementary Figures S13 and S14 and File S11). In contrast, the regular virome of each dataset was relatively unique with very low amounts of overlap (≤3) between datasets (species and genus shows a similar pattern). Interestingly, the highest overlap for species in the dark virome was between our dataset and (Ladd *et al.* 2017; 13), one of which is similar to RDRP viruses, although the contigs in Ladd’s data were not similar to the velvet tobacco mottle virus in our dataset (Supplementary Figures S15 and S16 and File S12).

In addition to comparing datasets using taxonomy, we also compared contigs between datasets for nucleotide similarity (>70%) using LAST (Supplementary File S1 for methods). We found that in general, datasets in the regular biome with the largest amount of contigs have the most overlap. Unsurprisingly, in the dark biome we observed less overlap by nucleotide similarity and that our RDRP contig does not share nucleotide similarity with contigs from any dataset. In addition, we also compared the nucleotide similarity of double dark biome contigs and found there is not a large percentage of similar contigs between datasets (Supplementary File S13).

### Comparison of taxonomy by condition within ALS datasets

Finally, we looked for differences in ALS versus control samples for each dataset. In the Gagliardidataset, when we compared ALS patients with the *FUS* mutation to controls, we found three significant differences in BNC by species in the regular bacteriome (*Neisseria sp.*, *Pseudomonas sp.*, and *Sphingomonas sp.*) and one significant difference in BNC by genus in the dark bacteriome (*Photobacterium*). In ALS patients with mutations in *SOD1* compared with controls, we found two species significantly different in the regular bacteriome (*Hydrogenophaga crassostreae* and *Sphingomonas hengshuiensis)*. We did not find anything significant in sALS, or in ALS patients with *TARDBP* mutations with regards to genera/species count (regular or dark biome or viruses) for Gagliardi. We found no significant statistical differences between ALS and control samples for genus/species count of viruses/bacteria in the regular/dark biome for any of the remaining ALS datasets.

### Meta-analysis between datasets

**Figure 8:**
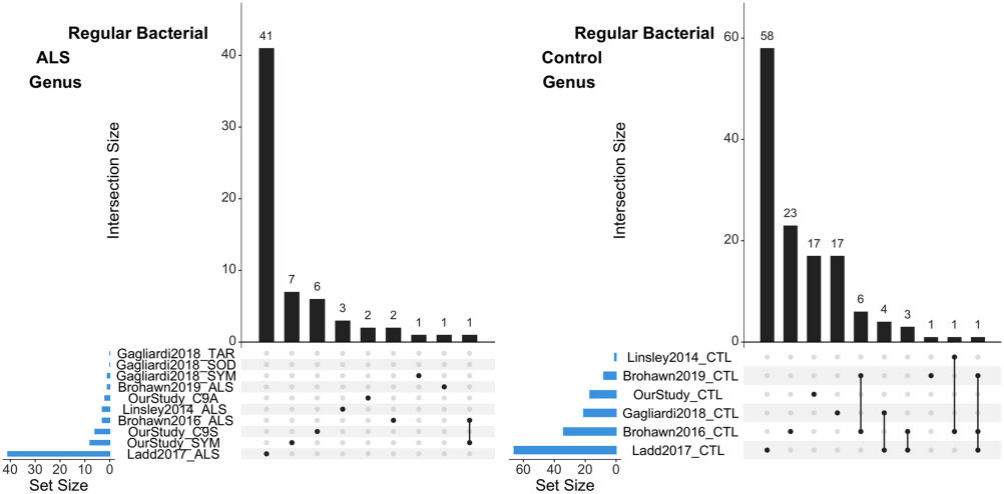
Upset plots of overlapping genus between datasets in the regular biome for ALS or controls. Upset plots are Venn diagram-like plots. A set refers to a contig that was assigned to a condition from a dataset. Each set is on a row with total amounts in a set as a blue bar plot on the left (ordered by set size). The black histogram on top shows the counts that are in the intersection of sets (a single dot for one dataset or connected dots for overlap of multiple datasets). (A). ALS contigs in the regular bacteriome. (B) Control contigs from the regular bacteriome.

Since our dataset and many others had few to no significant comparisons for ALS versus control groups within each dataset, a meta-analysis between datasets using this criteria would be difficult. As a second pass analysis we created a less stringent filtering method in order to compare the presence of microbes for each group between datasets (ALS *vs*. ALS; or controls *vs*. controls; Figure 8). We assigned a contig to a condition if ≥ 2 samples from that condition contain at least 90% of the summed NC (from all samples) to the contig. This filtering greatly reduced the number of comparable genus/species for each dataset and, for example, reduced the genus of the regular bacteriome in our dataset from 305 for all samples to 33 (SYM: 8, C9S: 6, C9A: 2, and CTL: 17; Supplementary File S14).

When we looked at ALS or control contigs in the regular bacteriome, the highest number of unique genus or species was from Ladd *et al.* (2017), and in general there was a small amount of overlap between datasets (≤1 for ALS or ≤ 8 for controls; Figure 8). When we looked at genus in the dark bacteriome we saw no overlap for ALS contigs and low overlap (≤1) between control conditions (species was similar; Supplementary Figures S17 and Supplementary 18 and File S14). In the regular virome there was no overlap between datasets and only our study (one contig from ALS) and Ladd *et al.* (2017; three from ALS and five from controls) had contigs that passed the filter (similar values for species). When we looked in the dark virome by genus there was no overlap between datasets, and our dataset had only one genus (*Sobemovirus* from controls) with the rest coming from Ladd *et al.* (2017; 18 from controls and 5 from ALS; Supplementary File S15). In conclusion, despite our conservative and loose approaches, we did not find any convincing evidence in ALS samples that the presence (or lack of presence) of an organism (or multiple organisms) was different compared with control samples.

## Discussion

We have created Mystery Miner to search for and quantify known and unknown microbes in RNA-seq datasets as a tool for researchers to study dysbiosis and identify infectious agents. We validated the pipeline by recovering and quantifying *Chlamydia* and SARS-CoV reads from RNA-seq datasets from intentionally infected cells. Interestingly, we also identified *Mycoplasma* reads in the *Chlamydia* dataset, suggesting that Mystery Miner may also be able to identify unsuspected bacterial infections or contamination. Next, we created a synthetic minibiome of two different *Chlamydia* species and SARS strains, along with six representative bacteria from the human microbiome to investigate the sensitivity of Mystery Miner with regards to species and strain detection and quantification of small fold changes at low coverage. We find that the pipeline is able to recover and quantify significant fold changes for the bacterial species but has difficulty distinguishing reads that come from highly related sequences. We also use published data to investigate the difference of polyA versus total RNA recovery of bacterial species in multiple tissues. Perhaps surprisingly, we did not see a consistent difference in the recovery of bacterial reads between the two types of RNA-seq libraries, considering that bacterial transcripts are not expected to be polyadenylated. However, it is well-recognized that polyA selection is imperfect, and libraries constructed from polyA-selected RNA routinely contain transcripts thought not to be polyadenylated (*e.g.*, rRNA). We also found increased recovery of bacterial species with globin RNA depletion in blood. This could be the result of an effective increase in read depth for bacteria when not sequencing globin, or an increase in contamination from the globin depletion step. We stress that our bioinformatic approach alone cannot distinguish between contamination and the true existence of microbial sequences in human tissue.

We then used Mystery Miner on a novel ALS blood dataset (Our Study) consisting of 8.64 × 10^9^ reads. This dataset was generated from whole blood total RNA that was depleted for both ribosomal and globin transcripts. It encompasses samples from controls, participants with a *C9ORF72* hexanucleotide expansion (symptomatic and presymptomatic), and *C9ORF72* negative ALS patients. We found no statistical difference in microbial sequence read coverage between controls and any class of ALS patients, examining either individual contigs or using various taxonomical binning approaches. We also did not detect any batch effects or obvious age or sex biases in the recovery of microbial reads (Supplementary Figure S8). Overall, we found no evidence of blood microbial sequences contributing to either *C9ORF72* negative ALS or symptomatic patients harboring the *C9ORF72* hexanucleotide expansion. However, ALS is a CNS disease, so our findings in these blood samples do not necessarily preclude a role for microbes in this disease.

A unique aspect of Mystery Miner is that it tracks nonhuman reads that do not have significant BLASTN hits in GenBank. We were intrigued by the identification of a large contig (>5 kb) in the dark biome of our ALS dataset that showed protein sequence similarity to RDRPS, the essential replicase of RNA viruses. To validate that this virus-like sequence was not an artifact of contig assembly or a contaminant introduced during library construction or sequencing, we used RT-PCR of the original patient samples to demonstrate that this sequence was present in positive samples identified through the RNA-seq analysis and not detectable in negative samples. We cannot extrapolate from this specific example to determine what fraction of the “dark” and “double dark” sequences represent true novel microbial sequences present in human blood, but we note that analysis of human cell-free blood DNA has reported the identification of thousands of novel bacterial sequences (Kowarsky *et al.* 2017). We suggest that Mystery Miner is a generally useful tool for the identification of novel microbial sequences in RNA-seq data.

To extend our analysis we processed publicly available blood and spinal cord ALS datasets through our pipeline. As observed in our dataset, library size generally correlated with number of bacterial contigs assembled and number of bacterial genera/species recovered. When the microbial sequences we found in our dataset were compared with the other datasets we found similar genera/species and, not surprisingly, larger datasets generally had greater overlap. One dataset (Ladd *et al.* 2017) yielded comparable recovery of bacteria and viruses for the regular biome but a far greater recovery bacteria and viruses in the dark biome compared with all the other datasets. This study used laser capture microdissection (LCM) to isolate cervical spinal cord motor neurons and had comparable read amounts per sample to other studies and was conducted in the same laboratory as two other studies (Brohawn *et al.* 2016). We are unsure why this dataset yielded a much larger dark biome compared with the other datasets. Potentially these differences are a byproduct of using LCM to acquire samples.

We then analyzed several publicly available ALS datasets for statistically significant differences between recovered microbial sequences in ALS and control samples. Only one dataset (Gagliardi *et al.* 2018) had any significant microbial sequence differences between control and ALS samples, specifically ALS patients with *FUS* or *SOD1* mutations. However, the sample number in this sub-study was small (*n* = 2–3), and in the case of the *SOD1* patients the excess microbial reads were in the control samples. In the absence of additional information (*e.g.*, batch assignments for the samples) it is difficult to conclude that these sequence/sample correlations are meaningful. Finally, we compared identified microbial sequences in the control and ALS samples across the datasets and did not identify any genera/species that were preferentially recovered in either sample type.

Using our bioinformatic analysis pipeline Mystery Miner, we have not identified an association between ALS pathology and sequences corresponding to known or unknown microbial species. However, there are intrinsic limitations in using “repurposed” RNA-seq data to assay tissue-associated microbial sequences, including the relatively small number of nonhuman reads (<1% of total) upon which the analysis is based. This limited sequence signal could preclude identification of rarer microbes. Perhaps more problematic is the possibility that contaminating sequences obscure true tissue-associated microbial sequences. Any candidate microbes identified using Mystery Miner that correlate with human phenotypes will necessarily require independent validation. Despite these limitations, we believe Mystery Miner will be a useful tool for future researchers investigating known and unknown microbes that could contribute to disease, as our analyses have shown it to be sensitive to bacterial/viral agents in sequencing data.

## Data availability

Raw sequencing data for Our Study dataset is available in the NCBI Sequence Read Archive under the accession number PRJNA715316.

All other datasets are publicly available, and all of the code used in this article is available at https://github.com/Senorelegans/MysteryMiner. Supplementary material available at figshare: https://doi.org/10.25387/g3.13315181.
